# Attachment of Human Epithelial Cells to an Anodized Titanium Surface

**DOI:** 10.3390/ma18143305

**Published:** 2025-07-14

**Authors:** Yoshihiko Akashi, Hayato Hashiguchi, Yoshitaka Yamaoka, Kei Nakajima, Katsutoshi Kokubun, Yoshiaki Shimoo, Kenichi Matsuzaka

**Affiliations:** 1 Department of Pathology, Tokyo Dental College, 2-9-18, Kandamisaki-cho, Chiyoda-ku, Tokyo 101-0061, Japan; akashiyoshihiko@tdc.ac.jp (Y.A.); hhashiguchi@tdc.ac.jp (H.H.); dentist.yamaoka@gmail.com (Y.Y.); nakajimakei@tdc.ac.jp (K.N.); kkokubun@tdc.ac.jp (K.K.); implant_zygoma@yahoo.co.jp (Y.S.); 2 MALO DENTAL & MEDICAL TOKYO, FUKUHARA GINZA F8, 7-8-10, Ginza, Chuo-ku, Tokyo 104-0061, Japan

**Keywords:** anodized titanium, attachment, human epithelial cells, abutment surface

## Abstract

The attachment of the oral epithelium to the abutment surface is crucial for the long-term success of dental implants. This study aimed to evaluate the attachment of human epithelial cells to anodized titanium surfaces. Anodized titanium discs were used as the experimental group, while machined titanium discs served as the control. Surface roughness and wettability were first measured for each group. Next, human epithelial cells were seeded onto each disc at a density of 4.0 × 10^4^ cells/cm^2^ and evaluated 3, 6, and 24 h later for cell proliferation, as well as mRNA expression and protein levels of laminin and integrin β_4_. Surface roughness was comparable between the two groups; however, wettability was significantly higher in the experimental group. Cell proliferation increased over time in both groups and showed no significant difference. Notably, the expression levels of both *laminin* and *integrin* β_4_ were significantly higher in the experimental group at 24 h. Furthermore, protein localization of laminin and integrin β_4_ was observed along the cell margins on the anodized surface. These findings suggest that anodization enhances epithelial cell attachment by promoting the expression and peripheral organization of key adhesion molecules.

## 1. Introduction

The importance of achieving osseointegration for the success of dental implants is well recognized. Now that osseointegration can be achieved with a high rate of success [1], the focus of dental implant systems has shifted to achieving long-term outcomes. However, even implants that have osseointegrated can still be lost due to causes such as peri-implantitis triggered by bacterial infection. Therefore, for dental implant systems, it is important not only to achieve osseointegration of the implant body but also to achieve “mucointegration” [2,3]—an attachment between the oral epithelium and the abutment, which is thought to create a barrier, called a “soft tissue seal,” for long-term tissue maintenance and stability.

Significant efforts have been made to optimize both osseointegration and mucointegration, with many studies focusing on surface modifications to enhance the cell attachment of the tissues surrounding the implant–abutment complex. Treatments such as ultraviolet (UV) irradiation, plasma treatment, and excimer laser treatment have been applied to titanium and zirconia surfaces. These treatments have been shown to impart superhydrophilic properties to the implant materials, thereby improving cell attachment with epithelial cells [4,5,6], fibroblasts [7,8,9], and osteoblasts [10,11].

Titanium is the most frequently used material in dental implant systems due to its mechanical properties and biocompatibility. Anodization has emerged as one type of surface modification that enhances the surface properties of titanium. Anodizing involves immersing the titanium in an electrolyte solution and applying a voltage to form a titanium oxide film on the surface, which improves the biocompatibility and mechanical strength of the implant [12,13], as well as its hydrophilicity. It has been reported that anodizing an implant surface contributes to improved osseointegration [14,15], but few studies have investigated its effect on the soft tissue response.

Anodized titanium has gained increasing attention for its potential to improve not only osseointegration but also soft tissue integration. Gulati et al. reported that nanotubular titanium dioxide structures formed by anodization promote the alignment and adhesion of gingival fibroblasts [16]. Building upon this, recent in vitro studies have demonstrated the enhanced adhesion and proliferation of both gingival epithelial cells and fibroblasts on anodized surfaces compared to untreated titanium [13,17]. Collectively, these findings support the concept that anodized abutments may contribute to the establishment of a more stable mucosal seal, which serves as a critical barrier to bacterial invasion. In a randomized controlled trial, Hall et al. compared machined and anodized abutments and found that anodization led to a sustained increase in keratinized mucosa height [18]. However, the underlying mechanism responsible for this result remains unclear.

Therefore, the purpose of this study was to evaluate the attachment of human epithelial cells to an anodized titanium surface.

## 2. Materials and Methods

### 2.1. Specimen Preparation and Surface Treatment

The titanium discs (13 mm in diameter and 0.5 mm in thickness) used in this study were made of a Ti alloy (TiAlV alloy, UNS R56401). In the control group, the surface of the discs remained untreated. In the experimental group, the disc surfaces were anodized. The anodization process created a golden-yellow-colored oxide layer with a controlled thickness of 150 nm on the surface of the titanium alloy through an electrochemical reaction, resulting in a highly durable and protective surface. The created titanium oxide layer was transparent and its golden-yellow appearance was an effect of thin-film interference.

### 2.2. Surface Roughness and Surface Wettability

The arithmetic mean surface roughness (Sa) was measured using a 3D laser measuring microscope (LEXT OLS4100, Olympus Corporation, Tokyo, Japan), with a length of 4 mm and a cut-off value of 0.8 mm, on three samples (*n* = 5).

The surface wettability of each of the five samples (*n* = 5) was characterized by contact angle measurements using a contact angle analyzer (Phoenix alpha P200, Meiwafosis Co., Ltd., Tokyo, Japan) 3 s after the application of a 4 mL droplet of distilled water.

### 2.3. Cell Culture

Cell culture experiments were performed using human HaCaT keratinocytes (Cytion, Eppelheim, Germany) [19,20]. HaCaT cells were cultured at 37 °C in a CO_2_ incubator (5%) in Dulbecco’s modified Eagle medium (DMEM) (high glucose) (Nacalai Tesque, Inc., Kyoto, Japan) with 10% fetal bovine serum (FBS) (Biowest, Nuaillé, France) and 1% penicillin/streptomycin (Gibco, Grand Island, NY, USA). The medium was renewed every 2–3 days. When the cells reached 80–90% confluence, they were detached using 0.25% trypsin ethylenediaminetetraacetic acid (EDTA) (Gibco, Grand Island, NY, USA) and subcultured at least three times. The subculture interval in this study was 3–4 days, depending on confluency. The titanium discs were placed in 24-well culture plates, and HaCaT cells were seeded onto each type of titanium disc at a density of 4.0 × 10^4^ cells/cm^2^.

### 2.4. Cell Proliferation Assay

HaCaT cells were seeded onto each type of titanium disc, and the viability of the attached cells was evaluated using WST-8-based colorimetry (Cell Counting Kit-8, Dojindo Laboratories, Kumamoto, Japan) at 3, 6, 24 h after seeding (*n* = 5). At each time point, the discs were washed twice with phosphate-buffered saline (PBS) to remove any unattached cells and were then moved to a fresh culture plate. Each culture plate was incubated with 40 mL of tetrazolium salt (WST-8) reagent and 400 mL of culture medium at 37 °C for 1 h, after which 110 mL of reaction solution was moved to a 96-well culture plate for evaluation. The amount of formazan product was measured using a microplate reader (Synergy H1, BioTek Instruments, Winooski, VT, USA) at 450 nm.

### 2.5. Quantitative Reverse Transcription Polymerase Chain Reaction (qRT-PCR)

HaCaT cells were seeded onto each type of titanium disc, and mRNA expression levels of the two epithelial cell attachment proteins, laminin and integrin β_4_, on each disc were measured using quantitative RT-PCR as follows (*n* = 3): At 3, 6, and 24 h, the discs were washed twice with PBS to remove any unattached cells, and HaCaT cells on the discs in each group were collected using a cell scraper. Total RNA was extracted from the HaCaT cells using an RNeasy Mini Kit (Qiagen, Hilden, Germany). The total RNA was then reverse-transcribed into cDNA using a ReverTra Ace^®^ qPCR RT Master Mix with gDNA Remover (Toyobo Co., Ltd., Osaka, Japan).

Quantitative RT-PCR was performed using a 7500 Fast Real-Time PCR System (Applied Biosystems, Waltham, MA, USA) using TaqMan^®^ Gene Expression Assays (Applied Biosystems, Waltham, MA, USA) to determine the mRNA expression levels of *laminin* (*Lamc2*, Hs00194345_m1) and *integrin* β_4_ (*Itgb4*, Hs00236216_m1). *Glyceraldehyde 3-phosphate dehydrogenase* mRNA (*Gapdh*, Hs02786624_g1) was used as an endogenous control. The reaction conditions consisted of an initial denaturation at 95 °C for 20 s, followed by 40 cycles of denaturation at 95 °C for 3 s and annealing/extension at 62 °C for 30 s. Each mRNA expression level was corrected based on the *Gapdh* mRNA expression level, and target gene expression levels were subjected to relative quantitative analysis.

### 2.6. Immunofluorescence Observations and Morphology

HaCaT cells were seeded onto each type of titanium disc for immunofluorescence observations. To observe the distribution of laminin and integrin β_4_, the cells were washed twice with PBS at 3, 6, and 24 h of cultivation and then were fixed in 4% paraformaldehyde in PBS (Fujifilm Wako Pure Chemical Corporation, Osaka, Japan) for 10 min at room temperature. The cells were then washed three times with PBS. Nonspecific binding was blocked with 1% goat serum for 30 min at room temperature.

The cells were incubated overnight at 4 °C with a primary antibody, either rabbit anti-laminin polyclonal antibodies (1:200 dilution, ab11575, Abcam, Cambridge, UK) or mouse anti-integrin β_4_ monoclonal antibodies (1:100 dilution, ab133682, Abcam, Cambridge, UK). After three additional washes with PBS, the samples were incubated for 30 min at room temperature with a secondary antibody, either Alexa fluor 555 goat anti-rabbit immunoglobulin G (IgG) (1:100 dilution, Invitrogen, Waltham, MA, USA) for laminin or Alexa fluor 555 goat anti-mouse IgG (1:100 dilution, Invitrogen, Waltham, MA, USA) for integrin β_4_, which were detected as red. FITC-conjugated phalloidin 488 (1:100 dilution, Invitrogen, Waltham, MA, USA) was used to observe actin filaments, which were detected as green. After five washes with PBS, a micro cover glass was mounted over each sample using the ProLong^TM^ diamond antifade mounting agent with DAPI (Invitrogen, Waltham, MA, USA) and they were left overnight at 4 °C. DAPI-stained nuclei were observed as blue.

These samples were imaged using a confocal laser scanning microscope (LSM880 with Airyscan NLO, Carl Zeiss AG, Oberkochen, Germany) equipped with dedicated software (ZEN 2 (Black edition), Carl Zeiss AG, Oberkochen, Germany).

### 2.7. Statistical Analysis

Statistical analysis was performed using one-way analysis of variance (ANOVA) at each time point, followed by Scheffé’s test for multiple comparisons. Data are presented as the mean ± standard deviation (SD). A *p*-value of <0.05 was considered statistically significant. All statistical analyses were conducted using Prism version 9 (GraphPad Software, San Diego, CA, USA).

## 3. Results

### 3.1. Surface Roughness and Surface Wettability

**Figure 1:**
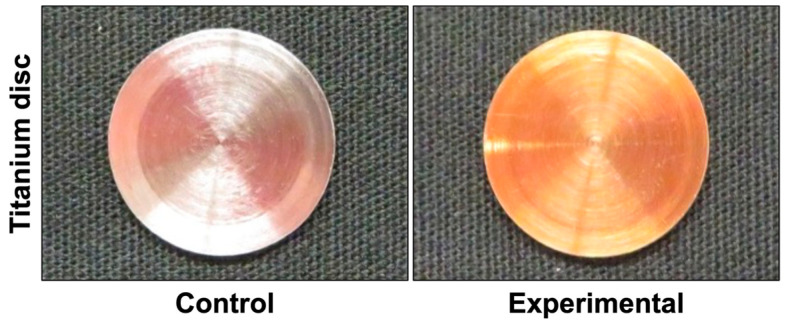
Macroscopic findings of titanium disc surfaces. Macroscopically, the titanium discs in the experimental group were gold in color, while the titanium discs in the control group appeared silver.

As shown in Figure 1, the titanium discs in the experimental group exhibited a gold coloration, while those in the control group appeared silver, indicating a visible difference in surface characteristics resulting from the anodization process.

**Figure 2:**
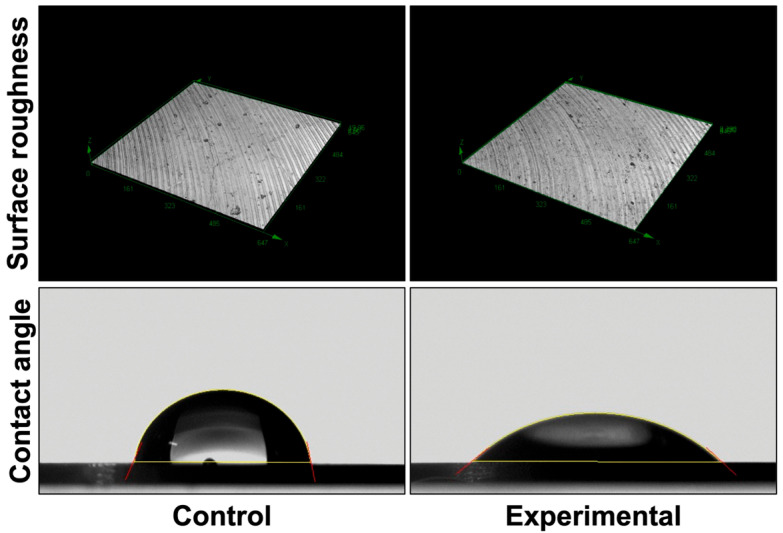
Surface roughness and contact angles of titanium discs. Using a 3D laser measuring microscope, the surfaces of the discs showed no difference in the surface texture between the experimental and the control groups. The surface of the titanium discs had a higher hydrophilicity in the experimental group compared to the control group.

As shown in Figure 2, observations using a 3D laser measuring microscope revealed that the surfaces of the discs in both groups exhibited grooves resulting from mechanical polishing, with no apparent difference in surface texture between the experimental and control groups. In addition, the surface of the titanium discs in the experimental group demonstrated higher hydrophilicity compared to those in the control group.

As shown in Table 1, the surfaces of the titanium discs in both groups were smooth, with Sa values of 0.1968 ± 0.0117 µm in the experimental group and 0.1830 ± 0.0226 µm in the control group, showing no significant difference.

As shown in Table 2, the contact angle of the titanium discs was significantly lower in the experimental group (36.2 ± 3.0°) than in the control group (72.3 ± 4.9°).

### 3.2. Cell Proliferation Assay

**Figure 3:**
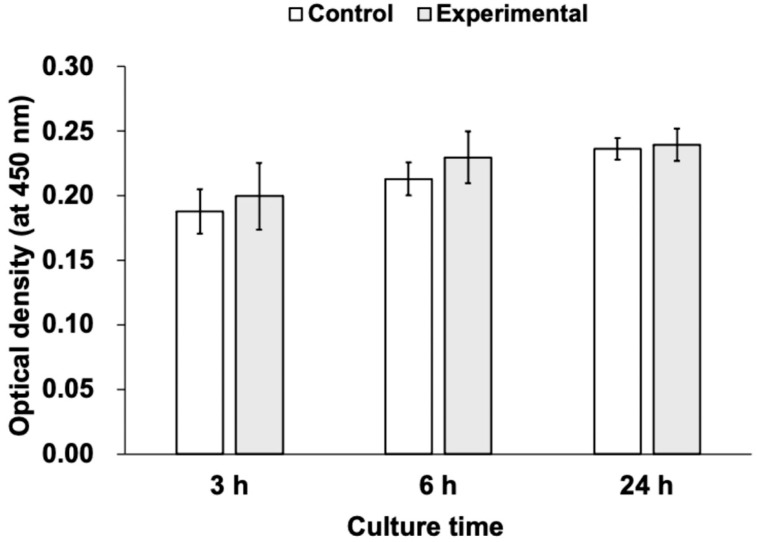
WST-8 values of the titanium discs. The cell proliferation activity, as measured by the WST-8 assay, increased over time, but no significant differences were observed between the two groups at any time period (*n* = 5).

Figure 3 shows the results of HaCaT cell proliferation at 3, 6, and 24 h of cultivation, as measured by the WST-8 assay. The cell proliferation of HaCaT cells, as measured by the WST-8 assay on each disc, increased over time up to 24 h, but there was no significant difference in WST-8 values between the two groups at any time point (Figure 3).

### 3.3. mRNA Expression (Quantitative RT-PCR)

**Figure 4:**
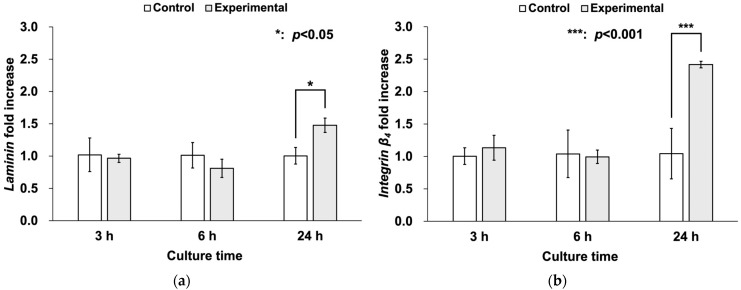
mRNA expression levels of *laminin* and *integrin* β_4_ (*n* = 3). (**a**) The mRNA expression levels of *laminin* were not significantly different between the groups at 3 and 6 h but were significantly higher in the experimental group compared to the control group at 24 h. (**b**) The mRNA expression levels of *integrin* β_4_ were not significantly different between the groups at 3 and 6 h but were significantly higher in the experimental group compared to the control group at 24 h.

The mRNA expression levels of *laminin* and *integrin* β_4_ in the HaCaT cells are shown in Figure 4a and 4b, respectively. The mRNA expression levels of *laminin* were not significantly different between the two groups at 3 and 6 h but were significantly higher in the experimental group compared to the control group at 24 h (Figure 4a). Similarly, the mRNA expression levels of *integrin* β_4_ were significantly higher in the experimental group compared to the control group at 24 h (Figure 4b).

### 3.4. Immunofluorescence Observations and Morphology

**Figure 5:**
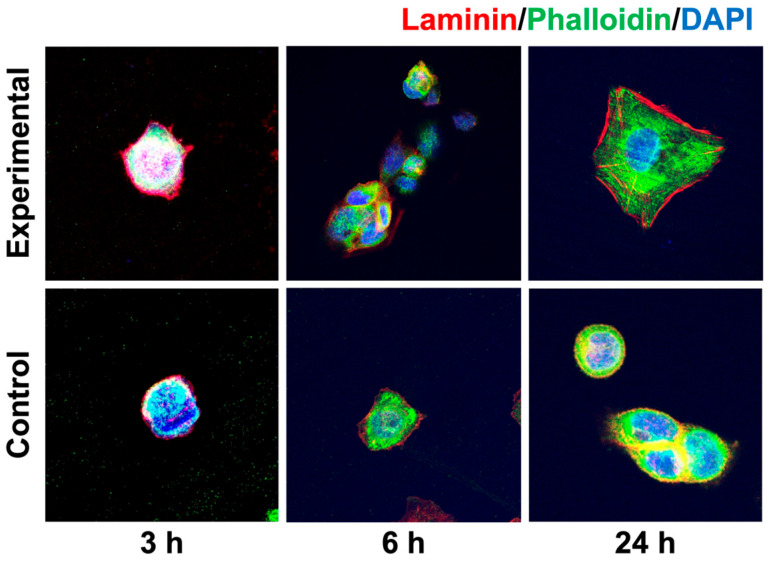
Immunofluorescence analysis of laminin. The immunofluorescence images of laminin revealed strong immunoreactivity along the edge of the cells in the experimental group at 24 h.

**Figure 6:**
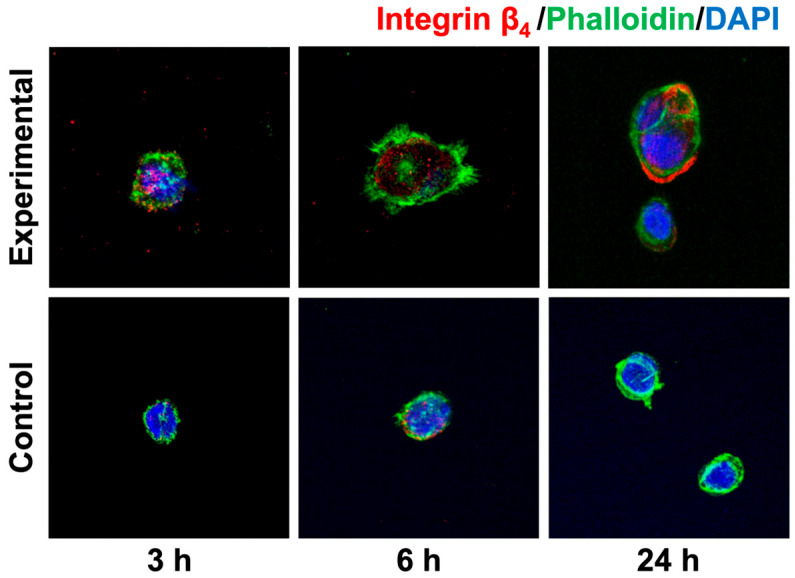
Immunofluorescence observations of integrin β_4_. Immunofluorescence images of integrin β_4_ revealed strong immunoreactivity along the edges of cells in the experimental group at 24 h.

Immunofluorescence images of laminin and integrin β_4_ are presented in Figure 5 and Figure 6, respectively. Immunofluorescent images of laminin showed immunoreactivity at the marginal regions of cells at 3, 6, and 24 h in both groups. In particular, strong immunoreactivity was observed along the edge of the cells at 24 h in the experimental group (Figure 5).

Immunofluorescence images of integrin β_4_ showed punctate (dot-like) immunoreactivity within cells at 3 and 6 h in the experimental group. In particular, strong immunoreactivity was observed along the cell edges in the experimental group at 24 h (Figure 6).

The immunofluorescence images of actin filaments showed immunoreactivity in the cytoplasm. In the experimental group, cells with a larger cytoplasm were observed, and some cells exhibited microspike-like extensions at their edges.

## 4. Discussion

This study investigated the attachment behavior of human HaCaT epithelial cells to an anodized titanium surface, evaluating the surface properties, cell proliferation, and expression and location of adhesion-related proteins.

Anodizing titanium is expected to improve its biocompatibility, aesthetics, corrosion resistance, etc. by forming a titanium oxide layer on the titanium surface. In dental implants, anodizing has been reported to promote osseointegration and to increase the success rate of implants [12,13,21]. It is known that the thickness of the oxide film formed varies depending on the voltage and time of anodizing. The thickness of the oxide film is related to the color tone of anodized titanium, and depending on the thickness, only certain wavelengths of light are reflected, resulting in a specific color tone [22]. This means that adjusting the thickness of the titanium oxide film on the abutment can improve aesthetic problems and is also clinically beneficial. The discs used in the experimental group were a uniform gold color, indicating that a titanium oxide film of uniform thickness was produced by the anodizing process. The uniform thickness of the titanium oxide film is likely the reason why there was no difference in the morphology of the disc surface visible by 3D laser microscopy before and after anodization, and why there was no significant difference in the Sa values. Consistent with these observations, Mussano et al. recently reported that the surfaces of machined and anodized titanium were both irregularly smooth and showed no significant morphological differences [23]. Similarly, Crenn et al. stated that anodizing alters the roughness at the nanometer scale but does not affect the micrometer-scale roughness, which depends on the material’s initial surface condition [17]. The present study also found no significant differences in microscale surface roughness, which is in agreement with these previous findings.

Additionally, anodized titanium surfaces have been reported to achieve superhydrophilicity due to hydrophilic functional groups generated on the surface [24]. In this study, the titanium discs in the experimental group exhibited enhanced surface wettability, which may contribute to improved epithelial cell attachment.

In this study, the proliferation of HaCaT cells measured by the WST-8 assay increased over time, but no significant differences were observed between the two groups. It has been reported that anodized titanium surfaces promote cell proliferation compared to untreated titanium surfaces [23,25,26]. However, there is one report that no significant difference in proliferation was observed [13]. In this study, cell proliferation was measured using the same anodized titanium, with a single cell type, and was only examined up to 24 h after seeding. Therefore, factors such as the cell type, the thickness of the oxide film, and the culture period may have influenced cell proliferation. For this reason, further investigation is necessary regarding the effects on cell proliferation.

A key finding was the significantly elevated expression of laminin and integrin β_4_ at the mRNA and protein level in the anodized group after 24 h. Laminins are major components of the extracellular matrix and are large proteins essential for the formation and function of basement membranes. Laminins are trimers consisting of three polypeptide chains, α, β, and γ, and play important roles in cell adhesion and tissue structural maintenance [27,28,29]. Integrins are a superfamily of cell adhesion receptors that act as adsorbed molecules that connect cells to the extracellular matrix and the cytoskeleton. Integrins are transmembrane αβ heterodimers, and at least 18 α and 8 β subunits are known in humans, generating 24 heterodimers [30,31,32]. In particular, integrin β_4_ is known to bind laminin in order to anchor epithelial cells to the basement membrane, to participate in the formation of hemidesmosomes, and to mediate cell attachment and migration [33]. Integrins also bind to actin via talin and vinculin, and are involved in cell migration, and their cooperation plays an important role in the assembly and maturation of cell adhesions [34]. In this study, the experimental group showed higher mRNA expression of *laminin* and *integrin* β_4_, as well as stronger immunoreactivity localized along the periphery of epithelial cells at 24 h, compared to the control group. These findings are consistent with previous reports. For example, Traver-Méndez et al. observed enhanced epithelial cell attachment on anodized implant collars, while Crenn et al. conducted a systematic review highlighting similar improvements in cell behavior on electrochemically anodized titanium surfaces [13,17]. Furthermore, actin ran through a wider cytoplasm and formed microspikes at the edges of cells in some areas. These observations suggest that a stronger attachment was achieved between the anodized titanium surface and the epithelial cells.

Atsuta et al. reported that, in normal gingiva, laminin is strongly expressed at the interface between the tooth and the epithelium, but in the peri-implant epithelium, laminin expression is limited to only one-third of the root side of the tooth [35]. This suggests that the coronal side of the peri-implant epithelium has poor adhesion and provides a weak biological barrier against bacteria. Therefore, we believe that strengthening the cell adhesion between anodized titanium and epithelial cells to a level close to normal is very important and beneficial in order to obtain an effective biological barrier against bacteria.

Several limitations must be acknowledged. The findings are based on in vitro data using a single immortalized epithelial cell line and a short observation period. No in vivo or clinical data were included to confirm whether the observed molecular changes would translate into enhanced biological function over time. Furthermore, the experimental setup did not incorporate the protective salt layer often applied to clinical-grade abutments, which may influence cell behavior in actual use. However, a previously published RCT demonstrated that anodization of abutment surfaces boosts keratinized mucosa height and improves soft tissue health [18].

From a clinical perspective, anodized titanium abutments hold promise for improving patient outcomes by enhancing soft tissue attachment, which may help reduce peri-implant inflammation, bacterial infection, and implant-related complications. In vivo evidence supports this potential, demonstrating that anodized abutments promote an increase in keratinized mucosal height, and strengthen the biological seal [36]. These findings are in line with our in vitro results, which suggest enhanced epithelial adhesion on anodized titanium surfaces. Strengthening this biological seal could contribute to the long-term stability and success of implant-supported restorations, aligning with the expectations of both clinicians and patients.

## 5. Conclusions

The results observed in this in vitro investigation suggest that the anodization of titanium discs enhances the adhesion of human epithelial cells. This enhancement is expected to strengthen mucointegration between the titanium abutment surface and epithelial tissue, potentially limiting peri-implantitis caused by bacterial infection, and preventing implant loss in the long term, helping to prevent long-term implant failure.

## Figures and Tables

**Table 1:** Surface roughness (Sa) values of titanium discs. Data represent the mean (±SD) (*n* = 5).

Titanium Disc	Surface Roughness (Sa) [μm]
Control	0.1830 ± 0.0226
Experimental	0.1968 ± 0.0117

**Table 2:** Contact angle of titanium discs. Data represent the mean (±SD) (*n* = 5).

Titanium Disc	Contact Angle [Degree]
Control	72.3 ± 4.9
Experimental	36.2 ± 3.0

## Data Availability

The original contributions presented in this study are included in the article. Further inquiries can be directed to the corresponding author.

## Abbreviations

ANOVA	analysis of variance
cDNA	complementary deoxyribonucleic acid
DAPI	4′,6-diamidino-2-phenylindole
DMEM	Dulbecco’s modified Eagle medium
EDTA	ethylenediaminetetraacetic acid
FBS	fetal bovine serum
FITC	fluorescein isothiocyanate
IgG	immunoglobulin G
mRNA	messenger ribonucleic acid
PBS	phosphate-buffered saline
qRT-PCR	quantitative reverse transcription polymerase chain reaction
Sa	arithmetic mean surface roughness
SD	standard deviation
UV	ultraviolet

## References

[1] Simão B.S., Costa D.D., Cristina M.T.C., Sotto-Maior B.S., Lana Devita R., de Carvalho J.J., da Silva Brum I. (2022). Observational study on the success rate of osseointegration: A prospective analysis of 15,483 implants in a public health setting. BioMed.

[2] Borie M., Lecloux G., Bosshardt D., Barrantes A., Haugen H.J., Lambert F., Bacevic M. (2020). Peri-implant soft tissue integration in humans—Influence of materials: A study protocol for a randomised controlled trial and a pilot study results. Contemp. Clin. Trials Commun..

[3] Elfarouk M. (2022). Implant mucointegration, a key to enhance osteointegration. J. Clin. Med. Case Rep. Rev..

[4] Nakhaei K., Ishijima M., Ikeda T., Ghassemi A., Saruta J., Ogawa T. (2020). Ultraviolet light treatment of titanium enhances attachment, adhesion, and retention of human oral epithelial cells via decarbonization. Materials.

[5] Kobune K., Miura T., Sato T., Yotsuya M., Yoshinari M. (2014). Influence of plasma and ultraviolet treatment of zirconia on initial attachment of human oral keratinocytes: Expressions of laminin γ_2_ and integrin β_4_. Dent. Mater. J..

[6] Kimura Y., Matsuzaka K., Yoshinari M., Inoue T. (2012). Initial attachment of human oral keratinocytes cultured on zirconia or titanium. Dent. Mater. J..

[7] Akashi Y., Shimoo Y., Hashiguchi H., Nakajima K., Kokubun K., Matsuzaka K. (2022). Effects of excimer laser treatment of zirconia disks on the adhesion of L929 fibroblasts. Materials.

[8] Razali M., Ngeow W.C., Omar R.A., Chai W.L. (2021). An in-vitro analysis of peri-implant mucosal seal following photofunctionalization of zirconia abutment materials. Biomedicines..

[9] Guo L., Smeets R., Kluwe L., Hartjen P., Barbeck M., Cacaci C., Gosau M., Henningsen A. (2019). Cytocompatibility of titanium, zirconia and modified PEEK after surface treatment using UV light or non-thermal plasma. Int. J. Mol. Sci..

[10] Krautwald L., Smeets R., Stolzer C., Rutkowski R., Guo L., Reitmeier A., Gosau M., Henningsen A. (2022). Osseointegration of zirconia implants after UV-light or cold atmospheric plasma surface treatment in vivo. Materials.

[11] Brezavšček M., Fawzy A., Bächle M., Tuna T., Fischer J., Att W. (2016). The effect of uv treatment on the osteoconductive capacity of zirconia-based materials. Materials.

[12] Guo T., Scimeca J., Ivanovski S., Verron E., Gulati K. (2023). Enhanced corrosion resistance and local therapy from nano-engineered titanium dental implants. Pharmaceutics.

[13] Traver-Méndez V., Camps-Font O., Ventura F., Nicolau-Sansó M.A., Subirà-Pifarré C., Figueiredo R., Valmaseda-Castellón E. (2023). In vitro characterization of an anodized surface of a dental implant collar and dental abutment on peri-implant cellular response. Materials.

[14] Villaça-Carvalho M., De Araújo J., Beraldo J., Prado R., Moraes M., Júnior L., Codaro E., Acciari H., Machado J., Regone N. (2021). Bioactivity of an experimental dental implant with anodized surface. J. Funct. Biomater..

[15] Kim M., Park K., Choi K., Kim S., Kim S., Jeong C., Huh J. (2015). Cell adhesion and in vivo osseointegration of sandblasted/acid etched/anodized dental implants. Int. J. Mol. Sci..

[16] Gulati K., Moon H.J., Kumar P.T.S., Han P., Ivanovski S. (2020). Anodized anisotropic titanium surfaces for enhanced guidance of gingival fibroblasts. Mater. Sci. Eng. C Mater. Biol. Appl..

[17] Crenn M.J., Dubot P., Mimran E., Fromentin O., Lebon N., Peyre P. (2022). Influence of Anodized Titanium Surfaces on the Behavior of Gingival Cells in Contact with: A Systematic Review of In Vitro Studies. Crystals..

[18] Hall J., Neilands J., Davies J.R., Ekestubbe A., Friberg B. (2019). A randomized, controlled, clinical study on a new titanium oxide abutment surface for improved healing and soft tissue health. Clin. Implant. Dent. Relat. Res..

[19] Boukamp P., Popp S., Altmeyer S., Hülsen A., Fasching C., Cremer T., Fusenig N.E. (1997). Sustained nontumorigenic phenotype correlates with a largely stable chromosome content during long-term culture of the human keratinocyte line HaCat. Genes Chromosom. Cancer.

[20] Boukamp P., Petrussevska R.T., Breitkreutz D., Hornung J., Markham A., Fusenig N.E. (1988). Normal keratinization in a spontaneously immortalized aneuploid human keratinocyte cell line. J. Cell Biol..

[21] Yao C., Webster T. (2006). Anodization: A promising nano-modification technique of titanium implants for orthopedic applications. J. Nanosci. Nanotechnol..

[22] Diamanti M., Curto B., Pedeferri M. (2008). Interference colors of thin oxide layers on titanium. Color. Res. Appl..

[23] Mussano F., Genova T., Laurenti M., Zicola E., Munaron L., Rivolo P., Mandracci P., Carossa S. (2018). Early Response of Fibroblasts and Epithelial Cells to Pink-Shaded Anodized Dental Implant Abutments: An In Vitro Study. Int. J. Oral. Maxillofac. Implant..

[24] Shibata Y., Suzuki D., Omori S., Tanaka R., Murakami A., Kataoka Y., Baba K., Kamijo R., Miyazaki T. (2010). The characteristics of in vitro biological activity of titanium surfaces anodically oxidized in chloride solutions. Biomaterials.

[25] Deng Z., Yu L., Kuang Y., Zhou Z., Li X. (2024). Highly ordered nanotube-like microstructure on titanium dental implant surface fabricated via anodization enhanced cell adhesion and migration of human gingival fibroblasts. Int. J. Nanomed..

[26] Corvino E., Pesce P., Mura R., Marcano E., Canullo L. (2020). Influence of modified titanium abutment surface on peri-implant soft tissue behavior: A systematic review of in vitro studies. Int. J. Oral. Maxillofac. Implant..

[27] Hohenester E. (2019). Structural biology of laminins. Essays Biochem..

[28] Timpl R., Brown J. (1994). The laminins. Matrix Biol..

[29] Engel J. (1992). Laminins and other strange proteins. Biochemistry.

[30] Mezu-Ndubuisi O., Maheshwari A. (2020). The role of integrins in inflammation and angiogenesis. Pediatr. Res..

[31] Takada Y., Ye X., Simon S. (2007). The integrins. Genome Biol..

[32] Van Der Flier A., Sonnenberg A. (2001). Function and interactions of integrins. Cell Tissue Res..

[33] Ogawa T., Tsubota Y., Hashimoto J., Kariya Y., Miyazaki K. (2007). The short arm of laminin gamma2 chain of laminin-5 (laminin-332) binds syndecan-1 and regulates cellular adhesion and migration by suppressing phosphorylation of integrin beta4 chain. Mol. Biol. Cell.

[34] Vicente-Manzanares M., Choi C.K., Horwitz A.R. (2009). Integrins in cell migration--the actin connection. J. Cell Sci..

[35] Atsuta I., Yamaza T., Yoshinari M., Goto T., Kido M.A., Kagiya T., Mino S., Shimono M., Tanaka T. (2005). Ultrastructural localization of laminin-5 (gamma2 chain) in the rat peri-implant oral mucosa around a titanium-dental implant by immuno-electron microscopy. Biomaterials.

[36] Dworan J., Aellos F., Grauer J.A., Fabbri G., Harder K.G., Boccardo S., Cuevas P.L., Dawid I., Vicini M., Helms J.A. (2025). Dynamics of Mucosal Integration of Machined versus Anodized Titanium Implants. J. Dent. Res..

